# Supplementary material for: The pseudobranch of jawed vertebrates is a mandibular arch-derived gill
Source: Development. 2022 Jul 11;149(13):dev200184. doi: 10.1242/dev.200184 (PMC9340550; doi:10.1242/dev.200184)
Supplement: Supplementary information [file develop-149-200184-s1.pdf]

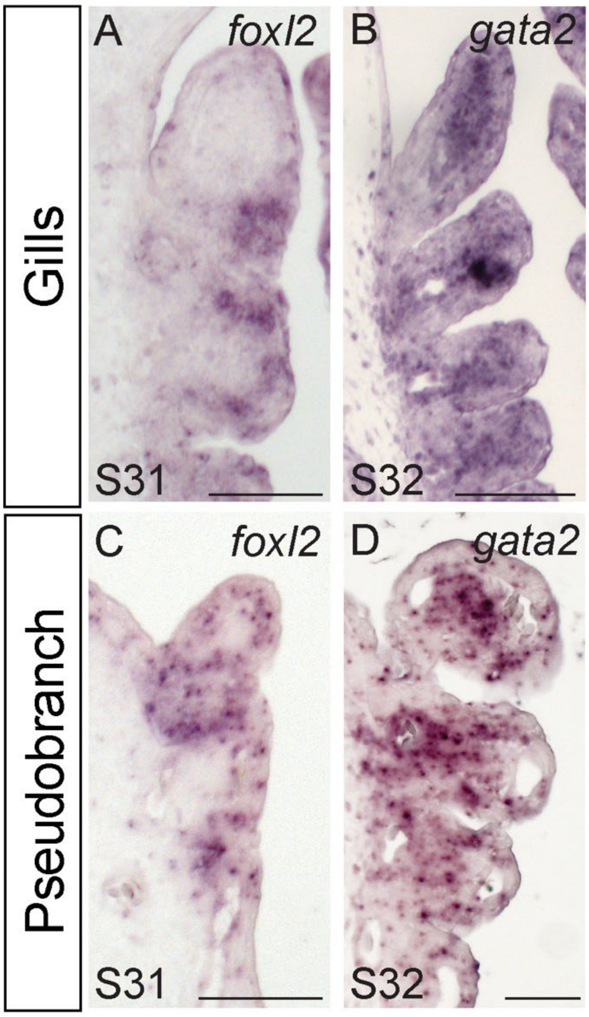

Fig. S1. Additional shared gene expression features of the gills and pseudobranch of the skate. (A) Expression of *foxl2* and **(B)** *gata2* in the gills of the skate. **(C)** Expression of *foxl2* and **(D)** *gata2* in the pseudobranch of the skate. Scale bars: 50µm.

Development • Supplementary information

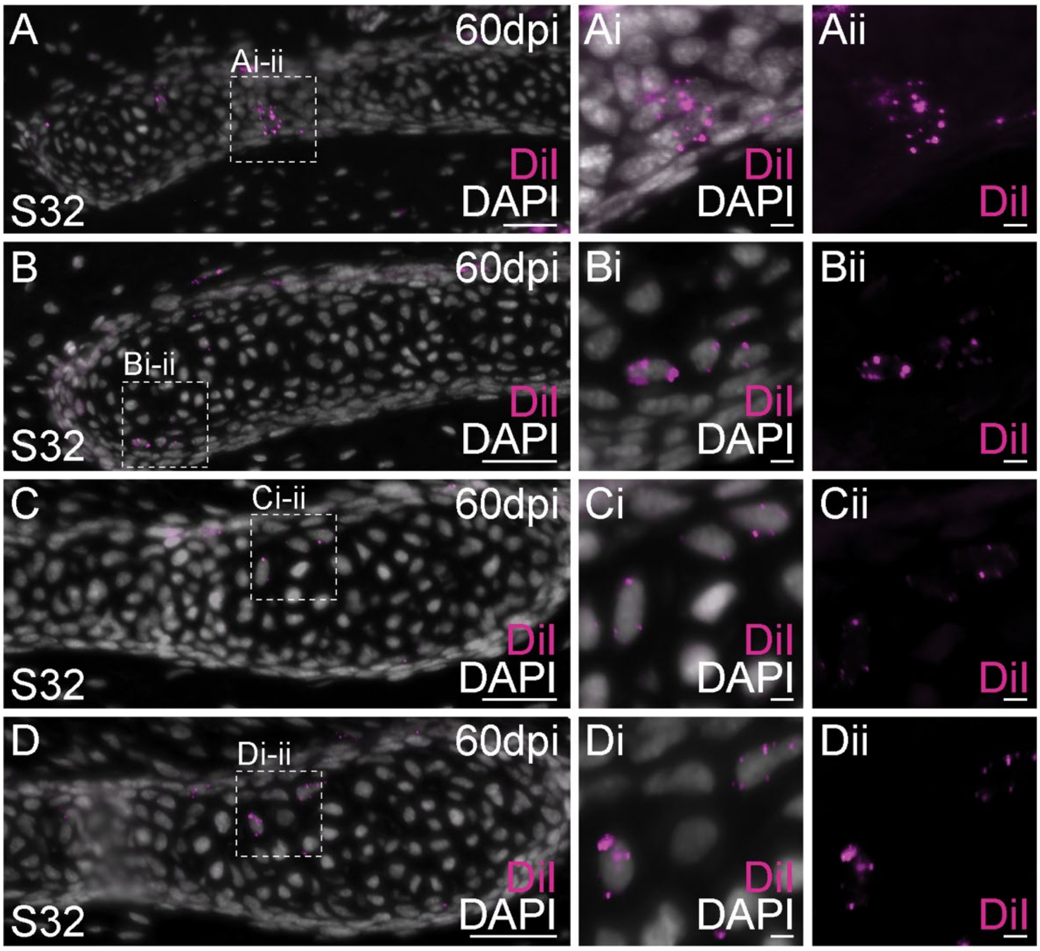

Fig. S2. Additional examples of CM-DiI-labelled chondrocytes, derived from mandibular arch GAER shh-responsive mesenchyme, in the skate spiracular cartilage. Scale bars: A-D=50µm, Ai/Aii-Di/Dii=5µm.

Development • Supplementary information

Heterodontus portusjacksoni (after De Beer, 1924)

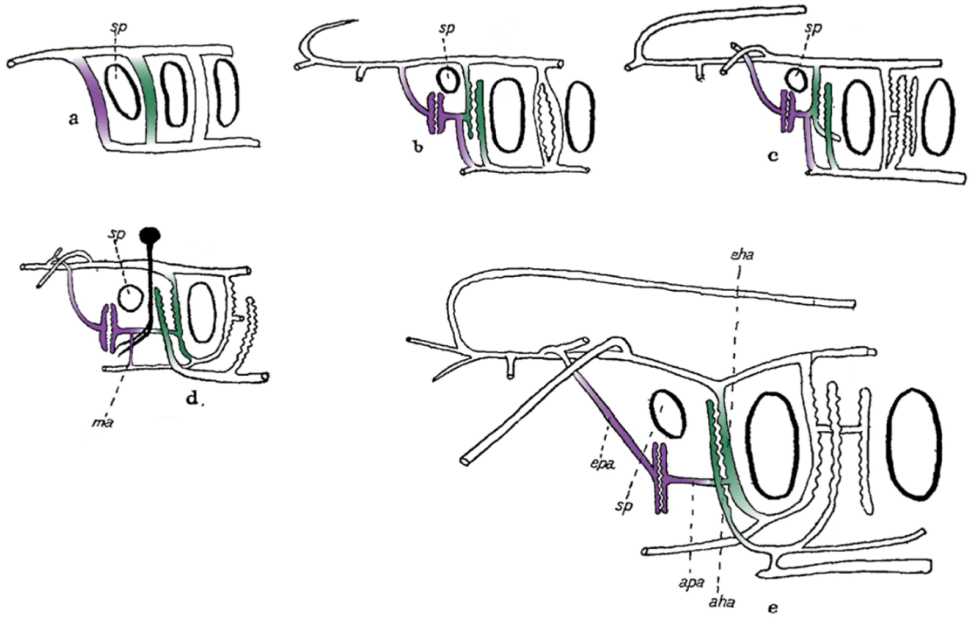

Squalus acanthias (after Scammon, 1911)

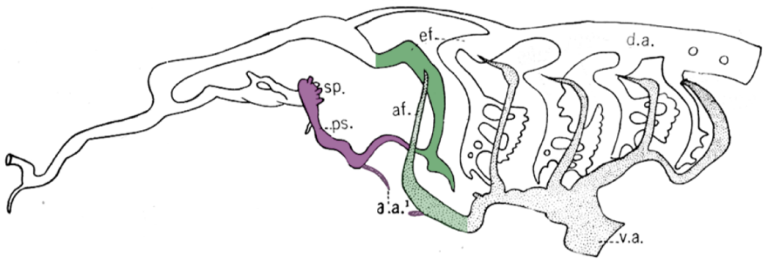

Fig. S3. Development and remodelling of pseudobranch vasculature in *Heterodontus portusjacksoni* and *Squalus acanthias*. At the top of the figure, embryological investigation of vasculature in *H. portusjacksoni* embryos after De Beer (1924). In **(a)**-**(e)**, mandibular arch vasculature is coloured purple, and hyoid arch vasculature is coloured green. **(a)** In an 11mm *H. portusjacksoni* embryo, the dorsal and ventral aorta are connected by distinct mandibular and hyoid arch blood vessels. **(b)** At 21mm, the spiracular hemibranch (future pseudobranch) has formed, the mandibular arch vessel has split into efferent and afferent pseudobranchial artery, and the hyoid arch vessel has split into efferent and afferent hyoidean arteries. The afferent pseuobranchial artery connects with the ventral aorta and, via a cross commissure, with the efferent hyoidean artery. **(c)** This vascular configuration persists at 25mm, and by **(d)** 45mm, the connection between the afferent pseudobranchial artery and the ventral aorta begins to breakdown. At this stage,

Development • Supplementary information

the remnant of the mandibular artery and the efferent hyoidean artery no longer communicate with the ventral aorta, and instead join the rudiment of the hypobranchial artery. **(e)** By 70mm, the remnant of the mandibular artery is gone, and the afferent pseudobranchial artery receives only oxygenated blood from the cross commissure with the efferent hyoidean artery. Selected abbreviations from the original paper: *aha*, afferent hyoidean artery; *apa*, afferent pseudobranchial artery; *eha*, efferent hyoidean artery; *epa*, efferent pseudobranchial artery; *ma*, mandibular artery; *sp*, spiracle. At the bottom of the figure, an image of embryonic cranial vasculature of *S. acanthias* by Scammon (1911). The pseudobranchial artery (purple) receives blood only from the efferent hyoidean artery (green), and remnants ($a.a^1$) of the recently degraded connection between the pseudobranchial artery and the ventral aorta are still visible. Selected abbreviations from the original paper: *af*, afferent hyoidean artery; *da*, dorsal aorta; *ef*, efferent hyoidean artery; *ps*, pseudobranchial artery; *sp*, spiracle; *va*, ventral aorta.

Development • Supplementary information
