# Supplementary material for: The pseudobranch of jawed vertebrates is a mandibular arch-derived gill
Source: Development. 2022 Jul 11;149(13):dev200184. doi: 10.1242/dev.200184 (PMC9340550; doi:10.1242/dev.200184)
Supplement: Reviewer comments [file develop-149-200184_review_history.pdf]

# The pseudobranch of jawed vertebrates is a mandibular arch-derived gill

Christine Hirschberger and J. Andrew Gillis
DOI: 10.1242/dev.200184

**Editor**: Cassandra Extavour

**Review timeline**

| | |
|---|---|
| Original submission: | 9 September 2021 |
| Editorial decision: | 3 February 2022 |
| First revision received: | 13 May 2022 |
| Accepted: | 14 June 2022 |


**Original submission**

First decision letter

MS ID#: DEVELOP/2021/200184

MS TITLE: The pseudobranch of jawed vertebrates is a mandibular arch-derived gill

AUTHORS: Christine Hirschberger and Andrew Gillis

Thank you for your patience as the review of this MS has taken much longer than we consider acceptable at Development. I apologize for this extreme delay. I have now received all the referees reports on the above manuscript, and have reached a decision. The referees' comments are appended below, or you can access them online: please go to BenchPress and click on the 'Manuscripts with Decisions' queue in the Author Area.

The overall evaluation is positive and we would like to publish a revised manuscript in Development, provided that the referees' comments can be satisfactorily addressed. Please attend to all of the reviewers' comments in your revised manuscript and detail them in your point-by-point response. In addition to the useful points and suggested raised by the external reviewer, having reviewed the MS as well myself I would like to ask you to include a few brief sentences in your introduction to make the concept of serial homology, and the challenges inherent in testing and falsifying hypotheses of serial homology, clearer to an audience of readers who are largely unfamiliar with the nuances of this concept. If you do not agree with any of their criticisms or suggestions explain clearly why this is so.

We are aware that you may currently be unable to access the lab to undertake experimental revisions. If it would be helpful, we encourage you to contact us to discuss your revision in greater detail. Please send us a point-by-point response indicating where you are able to address concerns raised (either experimentally or by changes to the text) and where you will not be able to do so within the normal timeframe of a revision. We will then provide further guidance. Please also note that we are happy to extend revision timeframes as necessary.

© 2022. Published by The Company of Biologists under the terms of the Creative Commons Attribution License (https://creativecommons.org/licenses/by/4.0/).

Reviewer 1

*Advance summary and potential significance to field*

I enjoyed reading this manuscript. It reports an impressive analytical and experimental approach to resolving a current disagreement in the contemporary literature regarding how to interpret the presence of a rudimentary gill (pseudobranch) on the first (or mandibular) arch of some extant bony and cartilaginous fishes: Does the presence of a pseudobranch represent the retention of a feature that was present on the first arch of early, jawless vertebrates that has been lost in some derived clades (and individual species) but retained in others, or has this feature appeared anew on the first arch following the evolution of jaws by cooption of developmental pathways (especially those that mediate development of gills) characteristic of more-posterior arches? The authors offer multiple sources of data in support of their overall conclusion, which comes down squarely in favor of the first alternative above.

*Comments for the author*

Methods are appropriate and the data appear solid. I have no serious concerns and cannot recommend any major changes. In the following list, I flag several minor errors and other items that need attention, including several in the References list that should have been caught and corrected before the manuscript was submitted. I also suggest the need for greater precision when discussing the implications of these results for the controversy described above, and also suggest an additional alternative explanation that reveals how difficult are such controversies to resolve definitively in the absence of a more complete and informative fossil record.
14. change “gill-arch like condition” to “gill arch-like condition.”
47-48. Something about this sentence left me confused, having just read the preceding paragraph. I think that “on the other hand” Is the culprit. Maybe change the last half of the sentence to something like, “… arch, however, remains contentious on other grounds.”
95-96. Please clarify “horizontal section.” In my experience, “transverse section” is more widely used and less ambiguous. However, the panel in B may actually be either a frontal or a parasagittal section.
100. Change “articular” to “articulation.”
134. Change to “then rinsed in…”
163. Insert “HCR amplification” before “hairpins”?
215. delete comma after “S29.”
216, 219, 232. Please clarify “posterior mandibular arch.” At first, I thought you were claiming that there are two mandibular arches, one anterior and one posterior, but I think you are referring to the posterior portion, or posterior face, of the mandibular arch (the latter wording is used on lines 225-226). Please remove this ambiguity and confusion.
223. Delete “entirely.”
334. Delete “of” after “rays.”
397. Insert hyphen between “shh” and “dependent.”
456-461, 617-626. The authors’ results provide compelling evidence in support of the hypothesis that the pseudobranch is serially homologous with gills on more-posterior arches, and this does challenge those in favor of co-option to account for the high degree of similarity, if not absolute identity, of the underlying developmental processes and relationships. But isn’t there a third alternative explanation for the presence of a pseudobranch, but one that accepts serial homology and shared developmental processes? What if gills that were once present on the ancestral mandibular arch were lost from that arch during the initial evolution of vertebrates but then reappeared on the first arch later during the evolution of jawed fishes? Reacquisition or re-evolution of lost features was once declared an evolutionary impossibility (e.g., Dollo’s Law), but with the production of more rigorous and reliable phylogenies, especially those derived from molecular data, more and more instances have been offered. I’m most familiar with those that involve amphibians (e.g., mandibular dentition in frogs, re-evolution of larvae in salamanders and caecilians). The problem with this third alternative is that, in the absence of additional direct evidence from the fossil record, it is very hard to distinguish from the interpretation favored by the authors, but also nearly impossible to distinguish from the co-option hypothesis. In any event, I urge the authors to consider offering at least the plausible existence of a third interpretation of the pseudobranch as described above.

© 2022. Published by The Company of Biologists under the terms of the Creative Commons Attribution License (https://creativecommons.org/licenses/by/4.0/). 

487-493. As someone who has taught comparative vertebrate anatomy many times, I am able to conjure a mental image of the relationships described here, but I'm not sure that many other readers will be able to understand this account. Would it be possible to include a simple figure that depicts these relationships, or to add them to figure 8?
540. Change "are associated" to "is associated."
615. Should "vertebrates" be qualified somewhat? For example, "earliest vertebrates"? "first vertebrates"? "earliest jawless vertebrates"? (This sidesteps the awkwardness of referring to a "mandibular arch" in primitively jawless vertebrates.)
615-617. Personally, I don't find the paleontological consensus to be very robust, mostly because of the difficulty of inferring many features of the earliest vertebrates from cyclostomes, which are recognized as being highly derived in many respects.
653. Why are names inside parentheses italicized? Also, Scyliorhinidae is misspelled.
671. Inconsistent style used for book titles. Compare this one with others in the Lit Cited, e.g. 748.
672. Likewise, inconsistent style used for capitalization of words in article titles. Compare this one with others in Lit Cited.
679-680. Reference not listed in alphabetical order.
696-713. Reference not listed in alphabetical order.
814. Reference not listed in alphabetical order.

---

**First revision**

Author response to reviewers' comments

We are very grateful for the excellent and constructive feedback that you and the other reviewer have provided, and we have revised our manuscript accordingly. Below, I outline a point-by-point list of the changes that we have made in response to your reviews.

***Editor comments to the authors***

***… I would like to ask you to include a few brief sentences in your introduction to make the concept of serial homology, and the challenges inherent in testing and falsifying hypotheses of serial homology, clearer to an audience of readers who are largely unfamiliar with the nuances of this concept.***

We completely agree that more context on this would be helpful for the largely developmental biology-focused audience of *Development*. We have therefore added a new paragraph to the introduction of our manuscript, which explains serial homology (with examples), and the challenges in testing hypotheses of serial homology from anatomical and developmental perspectives. We have also cited several key and foundational references in this paragraph, which readers might find helpful (and we have updated the reference list accordingly). The new paragraph reads as follows:

*"Patterns of serial homology may be recognised based on the apparent repetition or correspondence of anatomical features within an organism and are generally attributed to continuity or sharing of underlying developmental information (Van Valen, 1982; Roth, 1988; Wagner, 1989; Panchen, 1992). In many instances, serial homologues are arranged iteratively along the body, reflecting the serial repetition or re-deployment of a developmental mechanism along the embryonic axis (e.g., as with teeth, vertebrae, the paired appendages of jawed vertebrates or the wings and legs of arthropods - Van Valen, 1994; Ruvinsky and Gibson-Brown, 2000; Wagner, 2014; Bruce and Patel, 2020). But in some cases, serial homologues may arise by "co-option" of developmental mechanisms to spatially discontiguous locations within the body (e.g., as with the paired and median fins of jawed vertebrates - Freitas et al., 2006; Letelier et al., 2018). Importantly, serial homologues may be distinguished from structures within an organism that have converged on superficial anatomical similarity by their genesis via shared (whether iteratively or by co-option) or distinct developmental mechanisms, respectively (Hall, 2007). And while hypotheses of anatomical diversification of serial homologues over evolutionary time may be tested and falsified with palaeontological evidence, testing for shared developmental mechanisms as the basis of serial homology within extant taxa (and in the absence of fossil evidence) is*

© 2022. Published by The Company of Biologists under the terms of the Creative Commons Attribution License (https://creativecommons.org/licenses/by/4.0/). 

*inherently more difficult – the latter requiring a comparative analysis of embryonic context (e.g., tissue origin) and patterning mechanisms (e.g. tissue interactions, gene expression features and/or gene regulatory features), and ultimately an assessment of whether the weight of evidence points toward anatomical similarity arising via shared (i.e. conserved) or distinct generative pathways."*

***Reviewer 1 comments to the authors***

*14. change "gill-arch like condition" to "gill arch-like condition."*

Thank you, this has been corrected.

*47-48. Something about this sentence left me confused, having just read the preceding paragraph. I think that "on the other hand" Is the culprit. Maybe change the last half of the sentence to something like, "… arch, however, remains contentious on other grounds."*

Thank you, this has been corrected as per the reviewer's suggestion (and we agree that it sounds much better now!).

*95-96. Please clarify "horizontal section." In my experience, "transverse section" is more widely used and less ambiguous. However, the panel in B may actually be either a frontal or a parasagittal section.*

Thank you for pointing this out. We did, indeed, intend to use the term "frontal" section, and have adjusted this in the text.

*100. Change "articular" to "articulation."*

Thank you, corrected.

*134. Change to "then rinsed in…"*

Thank you, corrected.

*163. Insert "HCR amplification" before "hairpins"?*

Thank you, corrected.

*215. delete comma after "S29."*

Thank you, corrected.

*216, 219, 232. Please clarify "posterior mandibular arch." At first, I thought you were claiming that there are two mandibular arches, one anterior and one posterior, but I think you are referring to the posterior portion, or posterior face, of the mandibular arch (the latter wording is used on lines 225-226). Please remove this ambiguity and confusion.*

Thank you, we have changed these to "posterior face of the mandibular arch" for clarity.

*223. Delete "entirely."*

Thank you, corrected.

*334. Delete "of" after "rays."*

Thank you, corrected.

*397. Insert hyphen between "shh" and "dependent."*

Thank you, corrected.

© 2022. Published by The Company of Biologists under the terms of the Creative Commons Attribution License (https://creativecommons.org/licenses/by/4.0/).

*456-461, 617-626. The authors' results provide compelling evidence in support of the hypothesis that the pseudobranch is serially homologous with gills on more-posterior arches, and this does challenge those in favor of co-option to account for the high degree of similarity, if not absolute identity, of the underlying developmental processes and relationships. But isn't there a third alternative explanation for the presence of a pseudobranch, but one that accepts serial homology and shared developmental processes? What if gills that were once present on the ancestral mandibular arch were lost from that arch during the initial evolution of vertebrates but then reappeared on the first arch later during the evolution of jawed fishes? Reacquisition or re-evolution of lost features was once declared an evolutionary impossibility (e.g., Dollo's Law), but with the production of more rigorous and reliable phylogenies, especially those derived from molecular data, more and more instances have been offered. I'm most familiar with those that involve amphibians (e.g., mandibular dentition in frogs, re-evolution of larvae in salamanders and caecilians). The problem with this third alternative is that, in the absence of additional direct evidence from the fossil record, it is very hard to distinguish from the interpretation favored by the authors, but also nearly impossible to distinguish from the co-option hypothesis. In any event, I urge the authors to consider offering at least the plausible existence of a third interpretation of the pseudobranch as described above.*

Thank you for raising this point. We agree with the reviewer that atavism (or re-evolution) of a gill on the mandibular arch that was ancestrally present, but then also lost along the vertebrate stem, is an equally plausible explanation for our findings. And while our developmental data cannot distinguish between retention of an ancestral mandibular gill vs. atavistic re-evolution of a mandibular arch gill, I agree that it is important to explicitly acknowledge this possible explanation. So we have adjusted the first paragraph of our Discussion as follows:

*"We argue that these similarities may only reasonably be interpreted as products of serial homology, with jawed vertebrates either retaining (or possibly atavistically re-evolving) mandibular arch-derived gill structures that have been lost in extant cyclostomes, but that ancestrally formed iteratively from each pharyngeal arch (Fig. 8)."*

*487-493. As someone who has taught comparative vertebrate anatomy many times, I am able to conjure a mental image of the relationships described here, but I'm not sure that many other readers will be able to understand this account. Would it be possible to include a simple figure that depicts these relationships, or to add them to figure 8?*

This is an excellent point, and we appreciate that this anatomy is difficult for non-specialists to envision. We have therefore produced a new figure that illustrates remodelling of the mandibular and hyoid arch vasculature in cartilaginous fishes, based on the beautiful classical illustrations of De Beer (1924) and Scammon (1911). We have used illustrations of how the pseudobranchial artery is formed through development, and we have false coloured the relevant vessels to allow readers to more easily visualise the anatomy that we discuss in the text. As this is quite a large figure (with an extensive legend of descriptive text), we've opted to include this as a new Supplemental Figure 3 (rather than a main figure). But if you would prefer to have this in the main text, we are happy to move it there.

*540. Change "are associated" to "is associated."*

Thank you, corrected.

*615. Should "vertebrates" be qualified somewhat? For example, "earliest vertebrates"? "first vertebrates"? "earliest jawless vertebrates"? (This sidesteps the awkwardness of referring to a "mandibular arch" in primitively jawless vertebrates.)*

Thank you, and yes, we have qualified this to "stem-gnathostomes" rather than vertebrates (which, we agree, is imprecise in this context).

*615-617. Personally, I don't find the paleontological consensus to be very robust, mostly because of the difficulty of inferring many features of the earliest vertebrates from cyclostomes, which are recognized as being highly derived in many respects.*

© 2022. Published by The Company of Biologists under the terms of the Creative Commons Attribution License (https://creativecommons.org/licenses/by/4.0/). 

We completely agree. And although this “consensus” has been arrived it by the repetitive citation of a small number of highly influential works, we still feel that we should acknowledge this view (even if we don’t agree with it).

*653. Why are names inside parentheses italicized? Also, Scyliorhinidae is misspelled.*

Thank you, corrected.

*671. Inconsistent style used for book titles. Compare this one with others in the Lit Cited, e.g. 748.*

*672. Likewise, inconsistent style used for capitalization of words in article titles. Compare this one with others in Lit Cited.*

*679-680. Reference not listed in alphabetical order.*

*696-713. Reference not listed in alphabetical order.*

*814. Reference not listed in alphabetical order.*

Sorry for these inconsistencies. Given the format-free initial submission policy at *Development*, we were a bit relaxed about the organisation of our reference list. But the list has been carefully revised and edited to conform to *Development*’s style guide for this final submission.

Finally, while preparing this revision, a companion manuscript reveived positive reviews at eLife, and has now been revised and resubmitted. This preprint of this manuscript is available here: https://doi.org/10.1101/2022.03.15.484461. I have added a reference to this paper in the last paragraph of the Discussion section.

I hope that these changes thoroughly address all concerns. But should you wish to discuss any further changes to our manuscript, please feel free to get in touch – and thanks, again, for you time and consideration.

---

Second decision letter

MS ID#: DEVELOP/2021/200184

MS TITLE: The pseudobranch of jawed vertebrates is a mandibular arch-derived gill

AUTHORS: Christine Hirschberger and Andrew Gillis
ARTICLE TYPE: Research Article

I am happy to tell you that your manuscript has been accepted for publication in Development, pending our standard ethics checks. You'll see that both reviewers have only very minor corrective suggestions, which I suggest you incorporate in the proof for clarity.

Reviewer 1

*Advance summary and potential significance to field*

See my original review.

*Comments for the author*

In the new paragraph added to the Introduction (lines 98-119), I suggest adding "and their deployment" after "developmental mechanisms" on line 106.

© 2022. Published by The Company of Biologists under the terms of the Creative Commons Attribution License (https://creativecommons.org/licenses/by/4.0/). 

Please remove parentheses on lines 270-271 to eliminate the awkward conjugation in which "either" is outside the parenthetical statement but its partner "or" is inside it.

Reviewer 2

*Advance summary and potential significance to field*

The developmental origin of the pseudobranch is a long-standing question in evolutionary biology. While previous studies purported its developmental origin as non-mandibular pharyngeal arches, no one has comprehensively investigated its developmental process. This manuscript thoroughly tested gene expressions and cell lineages of the pseudobranch using histology, DiI labeling, and fluorescent in situ hybridization, indicating the developmental origin of the pseudobranch as the mandibular arch. Overall, the manuscript is clearly and concisely written with sufficient information, building fundamental knowledge in inquiries of pharyngeal arch derivatives.

The exhibited results and photos are exceptionally clear and support the authors' discussion, suggesting the non-canonical developmental origin of the pseudobranch. The text is very carefully and meticulously written leaving few rooms for scientific criticizing.

In line 288, the authors discussed reconfiguration of blood supply to the peudobranch during shark growth resolving the current confusion of the blood supply in comparative anatomy. The integration of the expertise of embryology into comparative anatomy in this context is outstanding.

*Comments for the author*

Minor concerns:
#159-165 – it's better to mention which tissues the authors labeled. From the figure, I guess DiI labeled both epithelial and mesenchymal cells.

© 2022. Published by The Company of Biologists under the terms of the Creative Commons Attribution License (https://creativecommons.org/licenses/by/4.0/).
